# Automatic method of analysis of OCT images in the assessment of the tooth enamel surface after orthodontic treatment with fixed braces

**DOI:** 10.1186/1475-925X-13-48

**Published:** 2014-04-22

**Authors:** Robert Koprowski, Monika Machoy, Krzysztof Woźniak, Zygmunt Wróbel

**Affiliations:** 1 Department of Biomedical Computer Systems, University of Silesia, Faculty of Computer Science and Materials Science, Institute of Computer Science, ul. Będzińska 39, Sosnowiec 41-200, Poland; 2 Department of Orthodontics, Pomeranian Medical University, al. Powstańców Wlkp. 72, Szczecin 70-111, Poland

**Keywords:** Image processing, Orthodontic, Measurement Automation, Segmentation, Tooth enamel

## Abstract

**Introduction:**

Fixed orthodontic appliances, despite years of research and development, still raise a lot of controversy because of its potentially destructive influence on enamel. Therefore, it is necessary to quantitatively assess the condition and therein the thickness of tooth enamel in order to select the appropriate orthodontic bonding and debonding methodology as well as to assess the quality of enamel after treatment and clean-up procedure in order to choose the most advantageous course of treatment. One of the assessment methods is computed tomography where the measurement of enamel thickness and the 3D reconstruction of image sequences can be performed fully automatically.

**Material and method:**

OCT images of 180 teeth were obtained from the Topcon 3D OCT-2000 camera. The images were obtained in vitro by performing sequentially 7 stages of treatment on all the teeth: before any interference into enamel, polishing with orthodontic paste, etching and application of a bonding system, orthodontic bracket bonding, orthodontic bracket removal, cleaning off adhesive residue. A dedicated method for the analysis and processing of images involving median filtering, mathematical morphology, binarization, polynomial approximation and the active contour method has been proposed.

**Results:**

The obtained results enable automatic measurement of tooth enamel thickness in 5 seconds using the Core i5 CPU M460 @ 2.5GHz 4GB RAM. For one patient, the proposed method of analysis confirms enamel thickness loss of 80 μm (from 730 ± 165 μm to 650 ± 129 μm) after polishing with paste, enamel thickness loss of 435 μm (from 730 ± 165 μm to 295 ± 55 μm) after etching and bonding resin application, growth of a layer having a thickness of 265 μm (from 295 ± 55 μm to 560 ± 98 μm after etching) which is the adhesive system. After removing an orthodontic bracket, the adhesive residue was 105 μm and after cleaning it off, the enamel thickness was 605 μm. The enamel thickness before and after the whole treatment decreased by about 125 μm.

**Conclusions:**

This paper presents an automatic quantitative method for the assessment of tooth enamel thickness. This method has proven to be an effective diagnostic tool that allows evaluation of the surface and cross section of tooth enamel after orthodontic treatment with fixed thin-arched braces and proper selection of the methodology and course of treatment.

## Introduction

Enamel is a tissue covering the dentine within the tooth crown. Its thickness in specific areas may exceed 1400 μm. Enamel is composed of about 98% of inorganic matter in the form of di-hydroxyapatite crystals. This chemical composition gives it unusual hardness. Organic compounds and water constitute the remaining 2% of enamel [1,2]. Orthodontic treatment concerning a significant part of the population is related to enamel when it comes to installing fixed braces. In this case, there occur changes in the structure of enamel after treatment with fixed orthodontic appliances which depend on the used orthodontic hooks. There are different methods of attaching fixed braces and treating the tissue after hook removal. There is also different efficiency of cleaning a tooth surface off the composite material residue after the removal of orthodontic hooks when using selected methods of enamel treatment [3].

For example, there are techniques of adhesive attachment of orthodontic hooks to the enamel surface which enable rapid development of orthodontic treatment with fixed braces [4-6]. This method is continuously being developed and adhesive systems as well as new types of brackets are being improved. Unfortunately, fixed orthodontic appliances cause a lot of doubts, because of their potentially destructive influence on the enamel [7,8] and others [9-12]. In [7], there are no statistically significant differences in volumetric changes after polishing between the different clean-up methods. However, sufficient clean-up without enamel loss was difficult to achieve. Enamel breakouts after debonding were detectable in 27% of all cases (mean depth of 44.9 mm). In [8], uncoated and precoated brackets exhibited similar debonding patterns. The debonding method tested in this study did not restore the original enamel surface, although there was no clinically relevant enamel damage. The use of coherence tomography in the areas of the mentioned enamel thickness or in the assessment of the strength of bonding is presented both in [9], with the use of a dental OCT handpiece, and in [10,12]. Therefore, there is a need for quantitative and not only qualitative assessment of tooth enamel in order to select the appropriate methodology and course of treatment as well as to assess the quality of enamel after treatment [13-16].

Evaluation of enamel quality can be done in many different ways. The known methods use an atomic force microscope (AFM) and a scanning electron microscope (SEM). It can also be evaluated using optical coherence tomography (OCT) [12]. This imaging method enables non-invasive structure visualization on the maximum enamel thickness and the desired tooth surface. The use of an optical tomograph having a wavelength of 840 nm to evaluate the enamel thickness is also interesting. For example, in [9] a central wavelength of 1310 nm and a spectral bandwidth of 47 nm were used for this purpose.

Unfortunately, at present it is relatively difficult to compare images taken at different time intervals and at different stages of treatment (diagnosis) [17-19]. The difficulty lies in automatic finding of the same areas or points on two or more images. Therefore, there is a need to propose a new automatic algorithm able to analyse OCT images acquired at different time intervals and quantitatively assess the treatment progress [20,21]. A profiled automatic algorithm for the analysis and processing of OCT images of tooth enamel, in particular, should enable to:

● quantify the changes in the enamel structure after treatment with fixed braces depending on the used orthodontic brackets, methods of attachment and tissue development after bracket removal,

● quantify the efficiency of cleaning a tooth surface off the composite material residue after the removal of orthodontic brackets when using selected ways of enamel development.

A description of the dedicated automatic algorithm is discussed in detail later in the paper.

## Material

**Figure 1:**
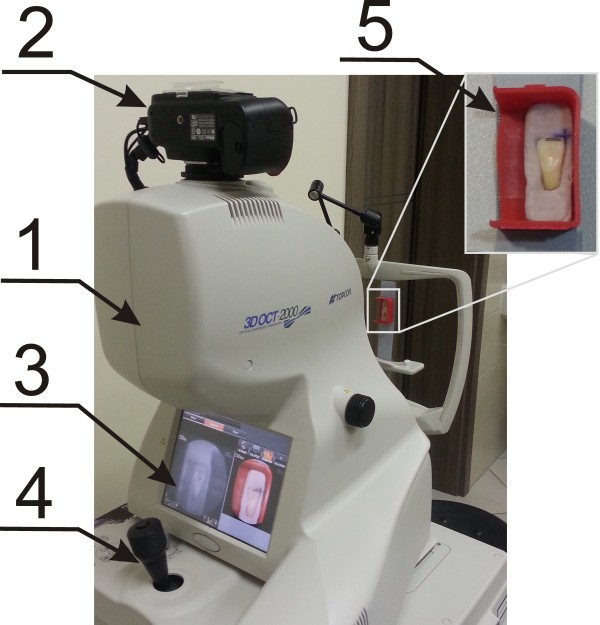
**Image showing the method of acquisition of OCT images of the teeth.** The following items are depicted: 1 - OCT tomograph, 2 - digital camera for taking images in visible light, 3 - screen of the tomograph, 4 - joystick enabling changes object position, 5 - method of attachment of the tooth in the device.

OCT images of 180 teeth were obtained from the Topcon 3D OCT-2000 camera - Figure 1. The images were obtained in vitro by performing sequentially 7 stages of treatment on each of the 180 teeth: before any interference into enamel, polishing with orthodontic paste, etching and application of a bonding system, orthodontic bracket bonding, orthodontic bracket removal, cleaning off adhesive residue. 7 OCT images were obtained at each of the 7 stages (the first image refers to a tooth without any interference).

All the teeth were extracted and then stored for 30 days in distilled water with 0.1% crystal thymol at room temperature, and then cleaned with pumice using a eraser (TopDental), rinsed with distilled water and dried with compressed air for 15 seconds. The composite material BluGloo (ORMCO, USA) was used to attach orthodontic brackets. The vestibular tooth surface was etched for 30 seconds with 36% phosphoric acid - Blue-Etch (CERKAMED, Poland), rinsed with distilled water and dried with compressed air. The adhesive system Ortho Solo (ORMCO, USA) was applied to the etched surface. A bracket was attached with the use of clamping tweezers for tooth enamel so that its centre was 3.5 mm below the edge of the occlusal surface, in the middle of the mesial-distal axis of the tooth. The test teeth were stored in water at 37˚C for 24 hours. Adhesive material residue was removed from enamel in two ways: by the use of a carbide cutter and by means of cup-shaped elastics OneGloss 0181 (Shofu Dental Corp., USA).

The acquired images (B-scans) had a resolution of *M* × *N* = 884 × 512 pixels (where *M* - number of rows, *N* - number of columns) where a single pixel covered the area of 5 × 11.7 μm at the colour resolution of 8 bits/pixel. For each patient *I* = 128 B-scans (every 47 μm) were obtained, which allowed for full 3D tooth image reconstruction. All data were analysed in a source format *.fda, *.fds. The data were anonymised and tooth extraction in all the patients was performed in accordance with the Declaration of Helsinki.

## Method

### Pre-processing

**Figure 2:**
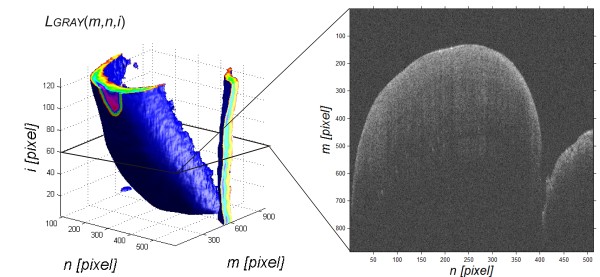
**Reconstruction of a sequence of images *L***_***GRAY***_**(*m,n,i*) for *M*** **×** ***N*** **×** ***I*** **= 884 × 512 × 128 pixels.** The image shows the result of reconstruction of an image sequence with a sample B-scan obtained for *i* = 60. The analysis aims at automatic determination of the edge on a sequence of OCT images (B-scans) in order to determine enamel thickness.

Pre-processing of images is related to automatic reading of a sequence of OCT images from a source file with the extension *.fds, thus obtaining a matrix of individual images. The read data also include information on the location of subsequent B-scans, their size, date of test, patient data and others. The sequence of images *L*_*GRAY*_(*m,n,i*) (Figure 2) thus obtained is further subjected to median filtering with a mask *h*_*1*_ sized *M*_*h1*_ × *N*_*h1*_ *× I*_*h1*_ = 3 × 3 × 3. The mask size was chosen arbitrarily taking into account the size of noise and artefacts entering the optical path. The filtered image *L*_*M*_(*m,n,i*) (where *m,n* are the coordinates of the rows and columns of the matrix *m*∈(1,*M*) and *n*∈(1,*N*)) is further subjected to preliminary transformations. These include determination of the enamel layer, measurement of its thickness in the entire area, comparison with other images of the same tooth and determination of the value of the proposed enamel quality coefficient.

### Processing

The filtered image *L*_*M*_(*m,n,i*) free of noise and artefacts is the basis for further transformations. These include determination of enamel thickness and imposition of two 2D images (C-scans) in order to compare changes in enamel thickness.

### Determination of enamel thickness

Determination of enamel thickness is related to determining the position of two enamel layers: outer *L*_*en*_(*n,i*) and inner *L*_*in*_(*n,i*) directly in the image *L*_*M*_(*m,n,i*). Determination of the outer layer *L*_*en*_(*n,i*) started from setting the upper limit of *L*_*ef*_(*n,i*) between the object (objects) and background:

(1) $$L_{\mathrm{ef}}\left(n,i\right)=\underset{m}{\text{min}}\left(L_{\mathrm{efm}}\left(m,n,i\right)\right)$$

where:

(2) $$L_{\mathrm{efm}}\left(n,i\right)=\left\{\begin{array}{ccc} m & \mathrm{if} & L_{M}\left(m,n,i\right)>p_{r}\\ N & \mathrm{other} & \; \end{array}\right)$$

and *p*_*r*_ – binarization threshold determined automatically – Otsu [22].

**Figure 3:**
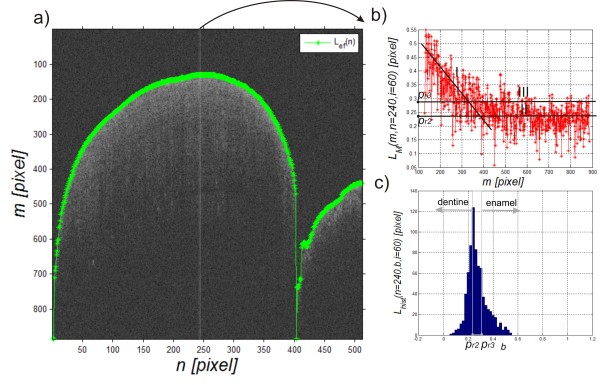
**Example of the result of the upper boundary analysis and selected parts of the analysis.** The image **a)** shows the result (in green) of automatic detection of the upper boundary *L*_*ef*_(*n,i*). The image **b)** shows changes in brightness for the selected A-scan (*L*_*M*_(*m*,*n* = 240, *i* = 60)) and the image **c)** shows the histogram on the basis of which the binarization threshold is selected in order to detect the enamel inner boundary.

The upper limit of the object *L*_*ef*_(*n,i*) is shown in green in Figure 3a. The presented method according to the equations (1) and (2) gives correct results in 100% of cases (for more than 20,000 images). This limit is used to determine the correct upper and lower boundaries of enamel (only a portion of the upper limit of objects *L*_*ef*_(*n,i*) is the appropriate outer boundary of the enamel *L*_*en*_(*n,i*)).

Detection of the enamel lower boundary *L*_*in*_(*n,i*) is related to the analysis of each column of the image *L*_*M*_(*m,n,i*) in the range from *L*_*ef*_(*n,i*) to the last row. Sample data (levels of brightness) are shown in Figure 3b and consist of two parts. Part I (Figure 3b concerns reduction of brightness of a fixed speed whereas part II refers to a constant brightness level. Part III is a shift of the mean (line II) by a standard deviation of the mean from this range to the top. The grey level in the range from *p*_*r2*_ to *p*_*r3*_ (II to III) is a place indicating tooth translucency reduction related to the end of the enamel layer and the start of another layer, namely dentine. The decision which of the threshold values should be taken as the correct one (in the range from *p*_*r2*_ to *p*_*r3*_) is connected in this case with a limit shift of about 70 pixels which corresponds to 70·5 = 350 μm. The value of this threshold can be automatically determined and clarified on the basis of the previous and subsequent columns and histogram analysis - Figure 3c. The threshold value *p*_*r2*_(*n*,*i*) (simplified to *p*_*r2*_) was calculated automatically based on the histogram:

(3) $$p_{r2}\left(n,i\right)=\underset{b}{\arg}\left(\underset{b}{\text{max}}\left(L_{\mathrm{hist}}\left(n,b,i\right)\right)\right)$$

where:

(4) $$L_{\mathrm{hist}}\left(n,b,i\right)=\sum_{m=1}^{M}L_{s}\left(m,n,b,i\right)$$

(5) $$L_{s}\left(m,n,b,i\right)=\left\{\begin{array}{ccc} 1 & \mathrm{if} & L_{M}\left(m,n,i\right)=b\\ 0 & \mathrm{other} & \; \end{array}\right)$$

**Figure 4:**
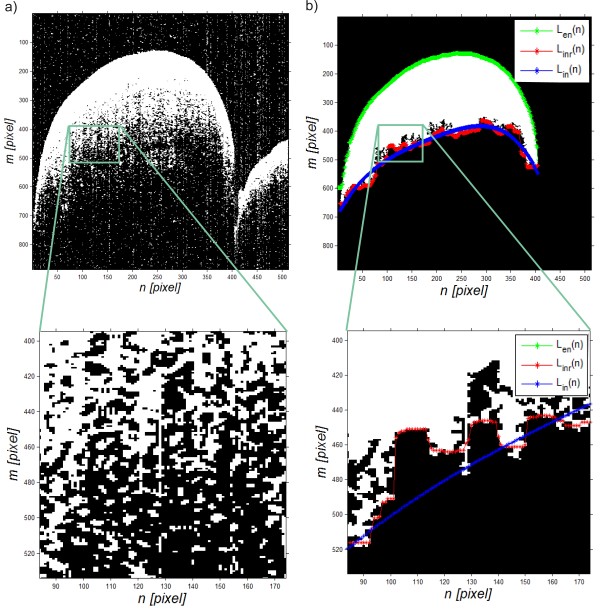
**Example of the binarization result of an OCT image and the results of analysis of inner and outer boundaries of the enamel.** The image **a)** shows problems that occur when determining the enamel inner boundary on a binary image whereas the image **b)** shows an example of the result of analysis based on the proposed algorithm. The outer boundary is marked in green, the inner boundary before filtration in red and the inner boundary after filtration in blue.

The obtained result, image *L*_*B*_(*m,n,i*) for the adopted binarization threshold, is shown in Figure 4a:

(6) $$L_{B}\left(m,n,i\right)=\left\{\begin{array}{ccc} 1 & \mathrm{if} & L_{M}\left(m,n,i\right)>p_{r2}\\ 0 & \mathrm{other} & \; \end{array}\right)$$

The binary image *L*_*B*_(*m,n,i*) is further subjected to median filtering with a mask *h*_*2*_ sized *M*_*h2*_ × *N*_*h2*_ *× I*_*h2*_ = 3 × 3 × 3. Then, the resulting image *L*_*MB*_(*m,n,i*) undergoes a morphological closing operation, i.e.:

(7) $$L_{C}\left(m,n,i\right)=\underset{\mathrm{SE}}{\text{min}}\left(\underset{\mathrm{SE}}{\text{max}}\left(L_{\mathrm{MB}}\left(m,n,i\right)\right)\right)$$

where *SE* – structural element sized 3 × 3.

The size of the structural element was chosen on the basis of measurements and average noise sizes (isolated ones) that make up clusters consisting of no more than 2 or 3 pieces. The next step involves filling holes and labelling the image *L*_*C*_(*m,n,i*). All further operations are carried out for each of the two-dimensional images for constant *i*, that is *L*_*C*_(*m,n*) (*L*_*C*_(*m,n,i = const.*))*.* Labelling enables to give a separate label (number) to each cluster of ones *L*_*W*_(*m,n*). In this case, it is possible to eliminate all of the clusters which do not have a maximum surface area. It results in the image *L*_*W*_(*m,n*) shown in Figure 4. In the next step, the inner enamel boundary *L*_*in*_(*n*) is determined as a 3rd order polynomial approximation of the course of the inner edge *L*_*inr*_(*n*) of an object in the image *L*_*W*_(*m,n*). An example of a polynomial takes the following form:

(8) $$L_{\mathrm{in}}\left(n\right)=-0.0001\cdot n^{3}+0.018\cdot n^{2}-3.21\cdot n+693.39$$

The outer edge of the enamel, *L*_*en*_(*n*) for short, is part of the previously defined edge of the object *L*_*ef*_(*n*) (for *i* = const.), i.e.:

(9) $$L_{\mathrm{en}}\left(n\right)=\left\{\begin{array}{ccc} L_{\mathrm{ef}}\left(n\right) & \mathrm{if} & \left(L_{\mathrm{ef}}\left(n\right)+1=1\right)\wedge \left(L_{\mathrm{ef}}\left(n\right)<N\right)\;\\ 0 & \mathrm{other} & \; \end{array}\right)$$

Based on the determined edges *L*_*ef*_(*n*) and *L*_*in*_(*n*), their difference is calculated:

(10) $$L_{d}\left(n\right)=L_{\mathrm{in}}\left(n\right)-L_{\mathrm{en}}\left(n\right)$$

**Figure 5:**
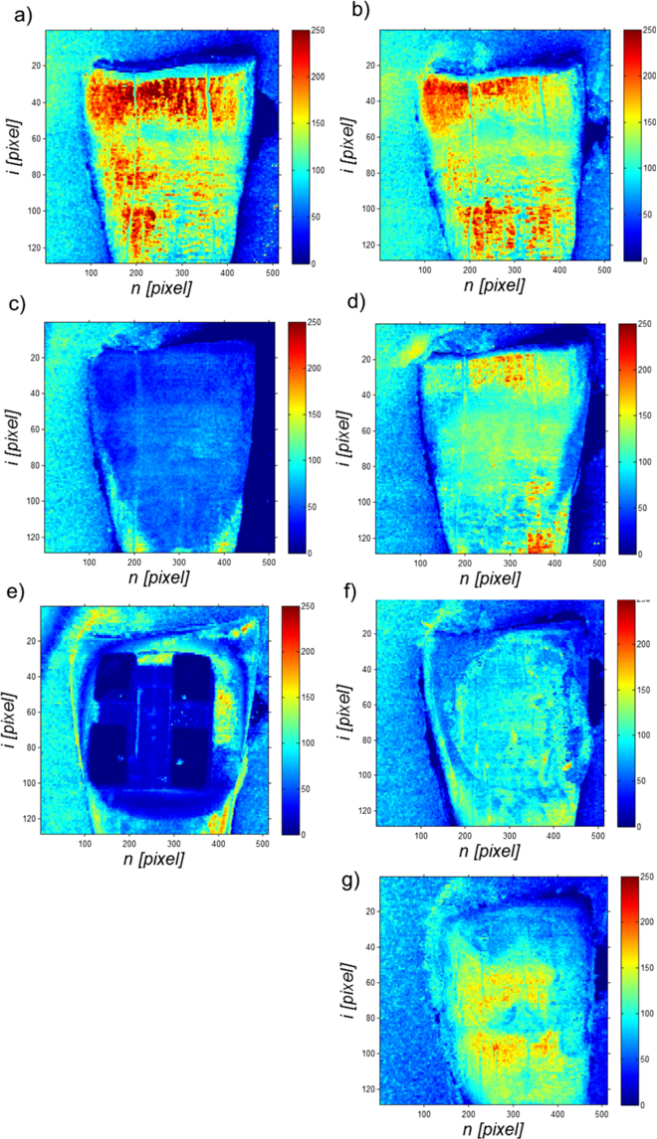
**Examples of results of enamel thickness measurement for the subsequent stages of tooth treatment.** It shows: **a)** enamel thickness before any interference *L*_*da*_(*n,i*), **b)** after polishing with paste *L*_*db*_(*n,i*), **c)** after etching *L*_*dc*_(*n,i*), **d)** after application of the adhesive system *L*_*dd*_(*n,i*), **e)** an orthodontic bracket *L*_*de*_(*n,i*), **f)** after bracket removal *L*_*df*_(*n,i*), **g)** after cleaning off adhesive residue *L*_*dg*_(*n,i*). These images then form the basis for the analysis of the impact of treatment methods and choice of orthodontic systems on changes in enamel thickness. The thickness is expressed in pixels for the artificial colour palette (ranges from blue to red, and passes through the colours cyan, yellow, and orange).

This difference is calculated for every *i* (for each B-scan), i.e. *L*_*d*_(*n,i*) forms a matrix of differences. The obtained matrices of differences for individual tooth treatment steps are shown in Figure 5 in the following order: the tooth before interference *L*_*da*_(*n,i*) - Figure 5a, after polishing with paste *L*_*db*_(*n,i*) - Figure 5b, after etching *L*_*dc*_(*n,i*) - Figure 5c, after the application of the adhesive system *L*_*dd*_(*n,i*) - Figure 5d, the orthodontic bracket *L*_*de*_(*n,i*) - Figure 5e, after orthodontic bracket removal *L*_*df*_(*n,i*) - Figure 5f, after cleaning off adhesive residue *L*_*dg*_(*n,i*) - Figure 5g. These matrices (from *L*_*da*_(*n,i*) to *L*_*dg*_(*n,i*)) are considered in the subsequent stages of analysis.

### Imposition of two 2D images

The images from *L*_*da*_(*n,i*) to *L*_*dg*_(*n,i*) show enamel thickness in various stages of tooth treatment. From a practical point of view, a change in thickness is important and, to be more exact, a change in enamel volume for each step. Therefore, it is necessary to propose a method of automatic imposition of individual images from *L*_*da*_(*n,i*) to *L*_*dg*_(*n,i*). Imposition of images, finding homogeneous points, is connected in this case with finding three values, namely a shift in the axis Δ*n*, a shift in the axis Δ*i* and zooming Δ*z*. In practice, due to the specificity of attachment used, the errors resulting from rotation did not exceed 1°. Because of this, it was assumed that there is no error in the images caused by rotation. The searched shifts in the axes Δ*n* and Δ*i* are further expressed in pixels whereas zooming Δ*z* is expressed as a percentage of the original image, i.e. 10% means a 10% zoom with respect to the reference image.

It is most convenient to find homogeneous points in standardized input images after median filtering and not in images showing different enamel thickness, i.e. (assuming that the denominator is different from zero):

(11) $$L_{T}\left(n,i\right)=\frac{\sum_{m=1}^{M}L_{M}\left(m,n,i\right)-{\text{min}}_{m,i}\left(\sum_{m=1}^{M}L_{M}\left(m,n,i\right)\right)}{{\text{max}}_{m,i}\left(\sum_{m=1}^{M}L_{M}\left(m,n,i\right)-{\text{min}}_{m,i}\left(\sum_{m=1}^{M}L_{M}\left(m,n,i\right)\right)\right)}$$

The first image *L*_*Ta*_(*n,i*) is here treated as the reference one, symbols of the other ones are analogous to *L*_*d*_(*n,i*), these are subscripts from ‘*b*’ to ‘*g*’ i.e. from *L*_*Tb*_(*n,i*) to *L*_*Tg*_(*n,i*). For each new shift and zoom relative to the reference image *L*_*Ta*_(*n,i*), the value of the match criterion *J* was calculated. The criterion *J* is the sum of absolute differences between the reference image *L*_*Ta*_(*n,i*) and the image being compared, e.g. *L*_*Tb*_(*n,i*). Therefore a point reaching the minimum and the values of Δ*n,* Δ*i* and Δ*z* for which it occurred are looked for. It is expressed as a percentage of changes with respect to the reference image, i.e., e.g. -10% means 90% of the reference image for a given magnification of the image (superscript is expressed as a percentage, e.g. for 120% relative to a standard, *L*_*Tb*_^*(100)*^(*n +* Δ*n,i +* Δ*i*). Therefore, the criterion *J* calculated for a pair of images *L*_*Ta*_ and *L*_*Tb*_ takes the following form:

(12) $$J=\frac{1}{I\cdot N}\sum_{i=1}^{I}\sum_{n=1}^{N}\left|L_{\mathrm{Ta}}\left(n,i\right)-L_{\mathrm{Tb}}^{\left(100\right)}\left(n+\Delta n,i+\Delta i\right)\right|$$

for Δ*n*∈(−40,40) pixels and Δ*i*∈(−40,40) pixels.

**Figure 6:**
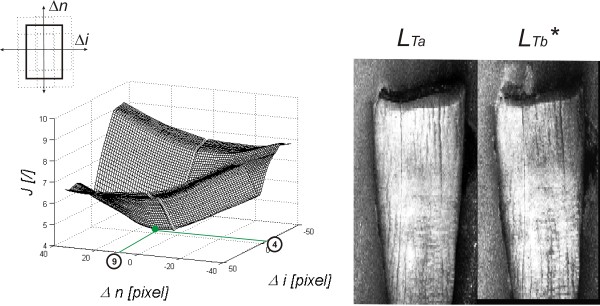
**Graph of changes in the criterion *J *for a sample pair of images*L***_***Tb***_^***(0)***^**(*n +*** Δ***n,i +*** Δ***i*) and*L***_***Ta***_**(*n,i*) and images after correction.** The value looked for in the graph is the minimum for which the values of shifts in both axes (Δ*n*, Δ*i*) and the zoom which gives the smallest value are read. In this case, the graph refers to a constant zoom of the image *L*_*Tb*_^*(0)*^ (0%) and for the example shown the following values were read from the graph: Δ*n =* 9 and Δ*i* = 4 pixels. In the left hand side of the image, there are the results obtained after shifting: images *L*_*Ta*_ and *L*_Tb_*.

The missing or redundant rows and columns in relation to *L*_*Ta*_(*n,i*) were removed or zero was written so that the numbers of rows and columns are identical. The position in both axes was changed every pixel, whereas zooming was carried out in the range from −20% to 20% every 10% relative to the reference image. The zoom for which calculations were made was marked as a superscript, e.g. if there is no image magnification then *L*_*Tb*_^*(0)*^(*n +* Δ*n,i +* Δ*i*). The obtained results, changes in the criterion *J* for different positions of the image *L*_*Tb*_^*(0)*^(*n +* Δ*n,i +* Δ*i*), in the absence of magnification, are shown Figure 6. After imposition of all the images and appropriate adjustment of the position of images from *L*_*db*_(*n,i*) to *L*_*dg*_(*n,i*) (after adjustment starting from *L*_db_^*^(*n,i*) to *L*_dg_^*^(*n,i*)), a region of interest (ROI) is determined. It has to meet two criteria: its total area must be within the matrix area and it must coincide with the contour of the tooth. The second criterion requires determination of the tooth area.

### Determination of the tooth contour

**Figure 7:**
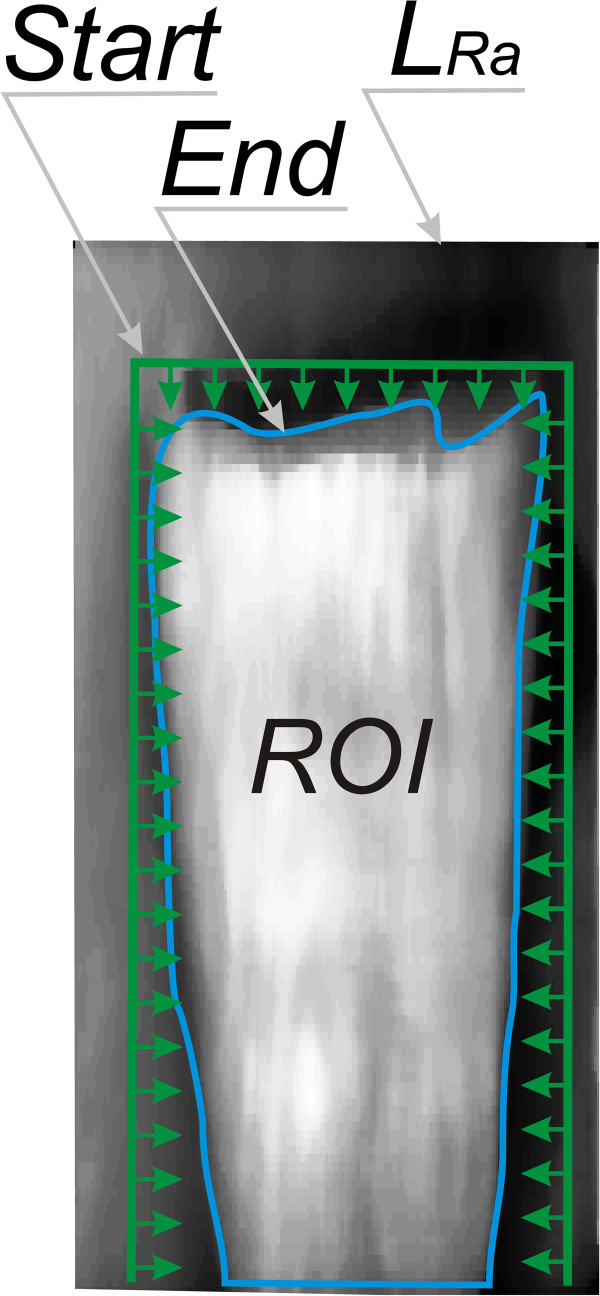
**Figure showing operation of the active contour method.** The input image *L*_*Ta*_ after median filtering (*L*_*Ra*_) is analysed using the active contour method. The standard whose size is independent of the size of the object is highlighted in green in the image. The blue shows the final stage of approximation - the area of ROI analysis.

Determination of the tooth contour is related to the analysis of one selected image *L*_*da*_(*n,i*) or *L*_*Ta*_(*n,i*). The other images from *L*_db_^*^(*n,i*) to *L*_dg_^*^(*n,i*) have contours in the same place, which results from their earlier imposition (finding homogeneous points). Just like in the case of imposition of images, the image *L*_*Ta*_(*n,i*) is easier to analyse than its corresponding image of the enamel thickness *L*_*da*_(*n,i*). In the first step, the image *L*_*Ta*_(*n,i*) is subjected to median filtering with a mask *h*_*3*_ sized *N*_*h3*_ *× I*_*h3*_ = 31 × 31 pixels. The mask size was selected on the basis of the average width of the tooth and the size of artefacts being the image noise. The output image *L*_*Ra*_(*n,i*) is the basis for determining the contour using the standard method and the active contour method. For the latter method, the following operating parameters were adopted: changes in the axes of rows and columns in the range of ±20 pixels and averaging the grey level in the areas sized 6 × 6 pixels on both sides of the analysed pixel. The algorithm stops work at a time when there is no evidence of differences between successive steps of bringing the contour closer to the object. The results are shown in Figure 7. The determined ROI is the basis for further analysis of the results. The resulting images from *L*_*ROIa*_ to *L*_*ROIg*_ contain information about the thickness of the layer only in the designated area of the tooth.

**Figure 8:**
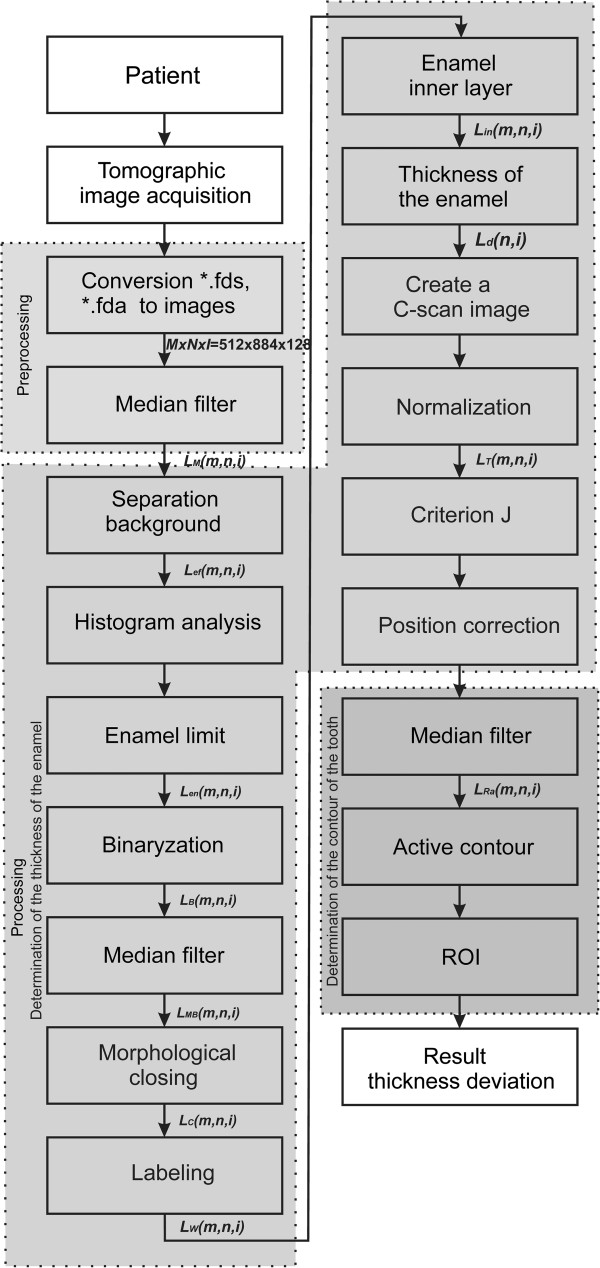
**Block diagram of the proposed method for image analysis and processing.** First, the input image is subjected to pre-processing (reading of data from the format *.fds, filtration) and then the enamel layer is recognized on subsequent B-scans. After that, C-scans obtained for subsequent phases of the enamel treatment are imposed on each other and the difference in enamel thickness in the ROI is measured. The last stage of processing involves comparison of changes in the thickness of enamel - its volume for the subsequent processing steps.

A block diagram of the proposed algorithm is shown in Figure 8.

## Results

The obtained images from *L*_*ROIa*_ to *L*_*ROIg*_ containing information about the thickness of the enamel layer are the basis for the calculation of mean thickness, standard deviation, and minimum and maximum values. The results are shown in Table 1, whereas Table 2 presents information about enamel thickness differences between the images. According to the adopted symbols from *L*_*ROIa*_ to *L*_*ROIg*_, these are: tooth before interference (index ‘*a*’), after polishing with paste (index ‘*b*’), after etching (index ‘*c*’), after application of an adhesive system (index ‘*d*’), an orthodontic bracket (index ‘*e*’), after removal of the orthodontic bracket (index ‘*f*’) and after cleaning off adhesive residue (index ‘*g*’). The differences confirm enamel thickness loss of 80 μm (enamel thickness at this stage is 650 ± 129 μm) after polishing with paste, loss of 435 μm (enamel thickness at this stage is 295 ± 55 μm) after bonding, an increase in the layer thickness of 265 μm (enamel thickness at this stage is 560 ± 98 μm) which is the bonding system. After the removal of the orthodontic bracket, the adhesive residue is 105 μm (enamel thickness at this stage is 455 ± 91 μm) and after removing the adhesive residue, the enamel thickness is 605 μm. The thickness of the enamel before and after the whole treatment decreased by about 125 μm.

**Table 1:** **Mean, minimum and maximum values of the enamel thickness in the ROI and standard deviation of the mean for the subsequent processing steps (according to the ROI designated in various stages - Figure** 5)

	***Subsequent processing steps [*** **μ** ***m]***						
***Parameter***	***L***_***ROIa***_	***L***_***ROIb***_	***L***_***ROIc***_	***L***_***ROId***_	***L***_***ROIe***_	***L***_***ROIf***_	***L***_***ROIg***_
***Mean***	730	650	295	560	80	455	605
***std***	165	129	55	98	135	91	140
***Min***	390	355	125	25	5	5	240
***Max***	1105	1015	515	820	775	665	860

**Table 2:** **Changes in the enamel thickness in the ROI for the subsequent processing steps (according to the ROI designated in various stages - Figure** 5**)**

	***Subsequent processing steps [*** **μ** ***m]***						
	***L***_***ROIa***_	***L***_***ROIb***_	***L***_***ROIc***_	***L***_***ROId***_	***L***_***ROIe***_	***L***_***ROIf***_	***L***_***ROIg***_
***L***_***ROIa***_	0	−80	−435	−170	−650	−275	−125
***L***_***ROIb***_	80	0	−355	−90	−570	−195	−45
***L***_***ROIc***_	435	355	0	265	−215	160	310
***L***_***ROId***_	170	90	−265	0	−480	−105	45
***L***_***ROIe***_	650	570	215	480	0	375	525
***L***_***ROIf***_	275	195	−160	105	−375	0	150
***L***_***ROIg***_	125	45	−310	−45	−525	−150	0

The results in Table 1 contain information about the minimum and maximum tooth enamel thickness. The differences in these values (minimum and maximum) result from actual variations in the enamel thickness in various locations of the tooth and the method errors. These errors are influenced to the greatest extent by the automatic selection of binarization thresholds *p*_*r2*_ and *p*_*r3*_ shown in detail in Figure 3 and the consequences in the binary image in Figure 4.

### Accuracy verification of the proposed method

**Figure 9:**
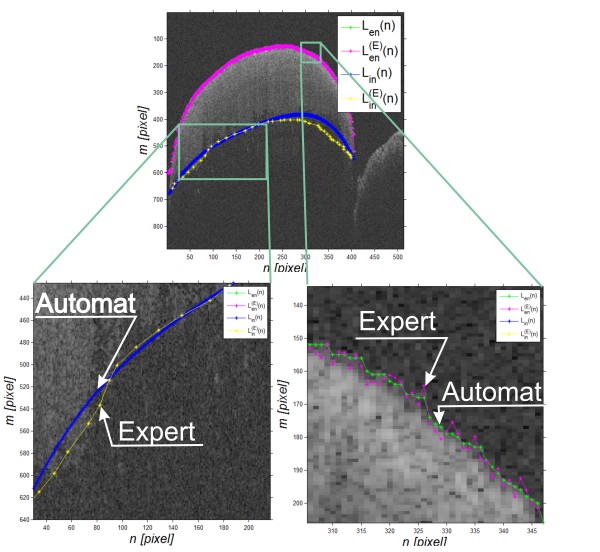
**Results of the automatic operation of the algorithm and the manual method of determining the enamel thickness.** The expert dentist marked two contours - the outer tooth contour *L*_*en*_^*(E)*^(*n*) (in magenta) and the enamel boundary *L*_*in*_^*(E)*^(*n*) (in yellow). On this basis and on the basis of the results of automatic analysis (*L*_*en*_(*n*) green, *L*_*in*_(*n*) blue) with the proposed algorithm, the measurement error was calculated per 20 teeth. This error did not exceed 15%.

The accuracy of the proposed method was assessed on the basis of the comparison of the results of enamel thickness measurements obtained with the proposed algorithm with independent measurements carried out by an expert. They involved manual marking of the enamel boundary by an expert on a tomographic image for 20 teeth (20·128 = 256 B-scans). In the case of the manual method, an expert dentist indicated two boundaries: the enamel boundary *L*_*in*_^*(E)*^(*n*) and the outer tooth boundary *L*_*en*_^*(E)*^(*n*) - Figure 9. On this basis, the enamel thickness was determined (analogously to (10)):

(13) $$L_{d}^{\left(E\right)}\left(n\right)=L_{\mathrm{in}}^{\left(E\right)}\left(n\right)-L_{\mathrm{en}}^{\left(E\right)}\left(n\right)$$

The measurement error, adopted as the measurement error of the proposed algorithm, was designated as:

(14) $$\delta_{d}=\frac{1}{N}\cdot \sum_{n=1}^{N}\frac{\left|L_{d}\left(n\right)-L_{d}^{\left(E\right)}\left(n\right)\right|}{L_{d}^{\left(E\right)}\left(n\right)}\cdot 100\%$$

For the analysed 20 teeth, the error* δ*_*d*_ did not exceed 15%. Individual results of the manual and automatic analyses are shown in Figure 9. In none of the analysed 20 teeth, the algorithm was mistaken by more than a few pixels when defining the outer tooth layer - *L*_*en*_(*n*). The contour of this layer was in each case very clearly visible and the differences between layer designation by an expert and the algorithm were visible only after zooming (in the lower part of the image Figure 9). In contrast, the enamel layer marked by the expert was only visible in selected ranges of columns. This situation, for layers *L*_*in*_(*n*) and *L*_*in*_^*(E)*^(*n*), is shown enlarged in Figure 9 at the bottom. Individual yellow points spaced apart by a few pixels indicated by the expert are visible. These points were further connected with segments. It was possible owing to information derived from anthropometric data, on whose basis the curvature of the contour and its possible variation per a single tooth are known. In summary, using the described algorithm, the measurement error δ_*d*_ does not exceed 15%.

### Comparison with other known methods

The method of analysis and processing of OCT images of tooth enamel developed by the authors can be compared to other known methods of analysis. These include:

● methods of analysis and processing of OCT images applicable in ophthalmology (e.g. recognition of retinal layers) and

● teeth image analysis carried out in visible light.

In the first group, there are methods which enable detection of individual layers in OCT images of the eye fundus [23-25]. The class of images is very similar to OCT images of tooth enamel. These methods work in a time comparable to that of the algorithm (from 10 to 200 ms/2D image) [26-28]. However, specificity of OCT images for the eye enables a significant simplification of the algorithm in the detection area of individual layers. This is due to a much better contrast between the layers. The advantage of OCT images of tooth enamel is the necessity of determining usually only one layer.

The other group of methods is closely related to dentistry but the ray spectrum range is significantly different (visible light). The acquired images do not allow for assessment of the thickness of any tissue, however, some of the methods of image imposition can be transferred to OCT imaging. An important difference is the possibility of using, in the case of visible light, RGB components which enable to distinguish a tooth from a handle and background. The applied active contour method brings correct results in both cases (visible light and OCT).

The forms of imaging, namely in vitro and in vivo, are also extremely important here. The differences are very interesting not only from the clinical point of view but also in terms of the algorithm of image analysis and processing. In the first case (in vitro), repeatable attachment of the tooth greatly facilitates image analysis - the segmentation of the tooth boundaries. In the other case (in vivo), the situation is more difficult because not only the background but also the mutual adjacent teeth arrangement change. In this case, there may also occur other problems with imaging such as placing the tomograph perfectly perpendicular to the tooth etc. However, preliminary tests confirm the usefulness of the proposed method, also in in vivo studies. The proposed segmentation process is resistant, in the area declared, to the impact of the background of the surrounding teeth (in the case of in vivo tests) on the results obtained. This is achieved owing to a segmentation method using the active contour. In this method, an important element is the contour of the tooth and not the background as may be the case in the method based on thresholding (binarization).

The presented method can also be compared to other methods of imaging of the enamel layer. As mentioned in the introduction, the known methods of tooth enamel analysis include assessment by means of an atomic force microscope (AFM) [29,30] and a scanning electron microscope (SEM) [31]. The class of images and the depth of imaging in these cases are quite different from the ones in OCT and typically confined to the outer enamel surface analysis – like, e.g., in [29]. A different class of images determines a different type of a profiled algorithm for image analysis and processing.

The other known methods of enamel thickness analysis do not enable automatic, quantitative measurement of the enamel thickness present in the ROI and automatic comparison of image groups. This is the case in [32], where comparisons between specific areas of the tooth enamel were made manually in OCT images. Automatic measurement was presented only in [33]. However, it concerns polarization sensitive optical coherence tomography (PS-OCT) and is not related to the problem of overlap of individual images in the subsequent processing stages of the tooth as shown in this paper.

### Summary

The paper presents the possibility of using the profiled algorithm proposed by the authors for the analysis of OCT images of tooth enamel. The algorithm of OCT image analysis and processing has the following features:

● works fully automatically - without any operator intervention,

● allows to quantify the changes in the structure of enamel,

● allows for quantitative assessment of the effectiveness of cleaning the tooth surface and the effectiveness of the use of selected methods of enamel development,

● analysis time of a sequence of 2D images does not exceed 5 seconds when using the Core i5 CPU M460 @ 2.5GHz 4GB RAM,

● the results of the mean thickness of the tooth enamel, minimum and maximum values as well as standard deviation are analysed automatically and saved to text files *.txt and Excel *.xls.

The presented algorithm is only one possibility of completing the task described in the paper. Automatic analysis of tooth enamel thickness provides a number of further possibilities. These include area analysis of enamel thickness (for each individual tooth area separately) and enamel texture analysis. Imaging and quantitative measurement of the enamel structure before installation of braces and after their removal enables to expose the tooth tissue damage extent depending on the used brackets and method of attachment. This makes it possible to deduce which brackets and what technique of their installation is the safest for tooth enamel.

The presented methodology does not fully exhaust the issue of automatic measurement of enamel thickness and the effect of the method of attachment of orthodontic brackets. The presented tool will be used in future work to assess the impact of multiple parameters. For example, the impact of the OCT settings on the results will be analysed. Moreover, the effect of time of in vitro studies on the result will be assessed. The analysis of reproducibility of results obtained in tomographic cameras at different wavelengths in the range from 600 to 1400 nm is equally interesting. An important aspect here will be confirmation of the universality of the proposed algorithm of image analysis and processing for different types of OCT devices or even the impact of the type of studies, in vitro or in vivo, on the process of segmentation.

It is also possible to modify the approach to the presented problem by using, for example, texture analysis [34], morphological approach [35], spectral analysis or other methods used for slightly different applications which, in practice, generate correct results [36-41]. In the future, the authors intend to perform and describe these methods, especially area and morphological analysis of the tooth enamel, by comparing the results obtained and measurement errors resulting from various adopted methods of analysis.

## Abbreviations

ROI: Region of interest; AFM: Atomic force microscope; SEM: Scanning electron microscope.

## Competing interests

The authors declare that they have no competing interests.

## Authors’ contributions

RK suggested the algorithm for image analysis and processing, implemented it and analysed the images. MM, KW, ZW performed the acquisition of the tomography images and consulted the obtained results. All authors have read and approved the final manuscript.
